# The perspectives of patients with depression toward esketamine, and the influence of their medication adherence on their viewpoints: a Saudi cross-sectional study

**DOI:** 10.3389/fpsyt.2025.1678119

**Published:** 2025-10-29

**Authors:** Ahmad H. Almadani, Ayedh H. Alghamdi, Fahad B. Alfahad, Abdullah S. Alibrahim, Abdulrahman I. Binbakhit, Ziyad B. Alenazi, Lama M. Alruwaili, Abdulrahman A. Alshahwan, Abdullah K. Muhnna, Mohammed A. Aljaffer

**Affiliations:** ^1^ Department of Psychiatry, College of Medicine, King Saud University, Riyadh, Saudi Arabia; ^2^ SABIC Psychological Health Research and Applications Chair (SPHRAC), Department of Psychiatry, College of Medicine, King Saud University, Riyadh, Saudi Arabia; ^3^ Department of Psychiatry, Eradah Mental Health Hospital, Al Kharj, Saudi Arabia; ^4^ College of Medicine, King Saud University, Riyadh, Saudi Arabia

**Keywords:** depression, esketamine, medication adherence, perspectives, Saudi Arabia

## Abstract

**Background:**

Nasal esketamine has demonstrated efficacy in the management of treatment-resistant depression and psychiatric emergency due to major depression. This study investigates acceptance and awareness of esketamine as a depression treatment option, focusing on factors that influence patients’ acceptance, including adherence to current medication regimens, regardless of prior esketamine exposure.

**Methods:**

This cross-sectional study surveyed 283 adults with depression using a questionnaire and the Medication Adherence Rating Scale (MARS-10).

**Results:**

52.3% of participants were willing to receive esketamine, and 51.2% preferred its weekly or biweekly dosing over daily antidepressants; 79.5% reported cost as a potential barrier. Common concerns included medication unavailability (59.7%), fear of addiction (50.5%), anticipated stigma (24.4%), and first-month dosing frequency (21.2%). Regarding adherence, 77.4% were nonadherent to their current psychiatric medication regimen. Adherence to the current regimen was higher among patients with previous esketamine use (*p* <.001) and among those who had someone to stay with them during and after treatment (*p* = .047).

**Conclusion:**

Patients are open to esketamine but have concerns that must be addressed. It also highlights non-adherence as a significant issue in patients with depression. These findings highlight the importance of patients’ education, family involvement, and logistical supports.

## Introduction

Depression is a significant mental illness worldwide and a leading cause of disability (1). In Saudi Arabia, the Saudi National Mental Health Survey reported a lifetime prevalence for depression of 3% for Saudi males and 9% for females (2). Depression can significantly affect interpersonal, social, and functional domains (3). Although depression is a treatable condition (4), relevant knowledge of available treatments in the general population remains limited in Saudi Arabia, as highlighted in Aljadani et al.’s 2021 study (5).

One of the novel treatments for depression is ketamine. Ketamine is a non-barbiturate anesthetic used for induction and maintenance of anesthesia (6, 7) and for procedural sedation, acute and chronic pain, post-operative pain, and hemodynamic instability (7, 8). Its water and lipid solubility enable intravenous, intramuscular, and intranasal administration with high bioavailability (6, 7). By targeting various receptor channels, ketamine produces a range of potent central nervous system effects (9). Its antidepressant potential was recognized in the late 1990s (10). Bremen’s 2000 study reported that intravenous ketamine produced antidepressant effects within hours (10). Subsequent studies have corroborated ketamine’s benefits (11, 12). For instance, a study demonstrated that intravenous ketamine rapidly alleviates suicidal ideation with minimal and transient side effects (11).

Multiple definitions of treatment-resistant depression (TRD) have been proposed and appear in the literature. One definition entails two failed trials of two antidepressants from different classes with documentation regarding adequate dosage, duration, and compliance (13). Another TRD definition, from the Thesa and Rush model, stages TRD across five levels, describing nonresponse to multiple medication classes, as well as to electroconvulsive therapy (13). Approximately 15–33% of patients with depression experience TRD, whereas conventional antidepressants fail to provide the desired effects, highlighting the need for innovative treatment (14). In 2019, after trials demonstrated its efficacy (15–22), the U.S. FDA approved intranasal esketamine for TRD, making a significant advance in psychiatric care (20). Esketamine can reduce depressive symptoms significantly within 24 hours (19) and rapidly decrease suicidal ideation in acute settings, as shown in a randomized controlled trial by Canuso et al. (19). Another study corroborated this anti-suicide effect of nasal esketamine spray, particularly among patients with a history of prior attempts (22). However, its use is associated with side effects such as dissociation, dizziness, and the potential for abuse (23). The intranasal administration of esketamine is non-invasive and convenient, which may enhance patient adherence; it is favored over placebo due to its ease of use and rapid absorption (24). Ahmed et al. (2023) recently reviewed the latest clinical evidence and guidance on esketamine nasal spray for TRD, including its potential to meet unmet patient TRD needs in the Arabic Gulf countries (25). Other studies have also explored the topic. For instance, in the TRANSFORM-2 trial, combining esketamine with either a selective serotonin reuptake inhibitor (SSRI) or a serotonin norepinephrine reuptake inhibitor (SNRI) demonstrated improvements in depressive symptoms over a four-week trial versus a placebo (17). In another study, the SUSTAIN-2 trial, the authors concluded that the long-term use of esketamine with either SSRI or SNRI maintained significant improvement in depressive symptoms and was well tolerated (21). Other studies have also indicated the long-term effectiveness and safety of esketamine. For instance, the SUSTAIN-3 study followed 1148 adults with TRD for over 6.5 years; participants exhibited clear improvements in depressive symptoms during the first four weeks of treatment. About half of the participants achieved remission. Side effects such as headache, dizziness, and dissociation were usually mild, resolved the same day, and rarely led patients to discontinue treatment (26). Further, a 2023 review of five studies concluded that esketamine is effective and safe for maintaining antidepressant effects (27). Nonetheless, it is also essential to highlight that myths surround the use of esketamine for TRD, as per Di Vincenzo et al.’s (28) clinical review, in which the authors discussed some of the myths related to TRD, including side effects and related issues.

Indeed, many aspects shape patients’ perceptions of the treatments. Research has revealed that patients’ perception of new treatments and whether they trust the healthcare system or hold certain beliefs about medications deeply inform their willingness to embrace these therapies (29, 30). When patients are engaged and participate in treatment decision-making, they are more likely to comply with the management plan, and the clinical outcomes tend to be better overall (29, 30). Jilka et al. (2021) explored patient perspectives on ketamine as a treatment for depression. They reported that many approached the treatment with skepticism; however, as patients became more familiar with the treatment, they shifted toward a positive outlook, recognizing the benefits of ketamine (31). Jilka et al. (2021) also emphasized that educating patients, carefully monitoring, and addressing concerns are key factors in ensuring patients’ acceptance and adherence, as well as the effectiveness of the treatment (31). Fairchild et al. (2020) investigated patient preferences for ketamine in TRD and found that patients place a high value on symptom improvement with ketamine and accept significant risks for these benefits (32). Koss (2021) used an educational video to inform the public and healthcare providers about the benefits and safety of ketamine infusion, resulting in positive engagement and increased awareness (33). Another study examined patients’ perceptions and attitudes toward esketamine, noting that active education engagement and positive changes in their symptoms during the treatment shape perceptions and attitudes, resulting in better adherence (34). Notably, in Saudi Arabia, no studies have directly assessed the perception of esketamine; however, Al-Shareef et al. (2024) conducted a study in the eastern region to examine psychotropic medications. The authors found that patients who had moderate to high knowledge had previous experience with psychotropic medication, had a family member who suffered from a psychiatric illness, or suffered from psychiatric illness themselves (35).

The present study was conducted in response to the considerable burden that major depressive disorder causes (1, 36) and the need to explore new treatments in the Saudi context. A third motive was to explore esketamine in Saudi Arabia. Collectively, the study aims to evaluate the acceptance and awareness of esketamine among patients diagnosed with depression and to examine the factors that may affect treatment acceptance, including adherence to current medication regimens, regardless of patients’ prior esketamine exposure.

## Material and methods

### Study design, setting, and participants

This quantitative cross-sectional study involved adult patients diagnosed with depression and related disorders at King Khalid University Hospital (KKUH), Riyadh, Saudi Arabia. Specifically, inclusion criteria included adults aged 18–65 and diagnosed with major depressive disorder, persistent depressive disorder, and other specified and unspecified depressive disorders. Exclusion criteria included those with communication barriers, and those diagnosed with bipolar affective disorder, primary psychotic disorders, depressive disorder secondary to another medical condition, depressive disorder secondary to substance, depressive disorder secondary to medications, and substance use disorder. Participants who did not meet the inclusion criteria or did not fully complete the study instruments were excluded.

The research team distributed the study instrument among the participants from mid-April to mid-June 2025 through an electronic survey. The research team contacted participants by phone to explain the study and invite participation; the survey link (sent by WhatsApp/email) was provided to those who agreed.

KKUH’s IT department identified 505 eligible patients. Based on this number, we calculated the sample size using raosoft.com (http://www.raosoft.com/samplesize.html) with a 5% margin of error and a 95% confidence level. The calculated sample size was 219 patients. We added an additional 25% to the calculated sample size to account for non-respondents, yielding a total of 273 participants.

### Survey instruments

The study instrument comprised three parts: (1) educational material about esketamine, (2) a questionnaire developed by the research team, and (3) the Medication Adherence Rating Scale (MARS-10).

The educational material summarized esketamine’s clinical indications, administration, pre-/post-treatment precautions, cost, and side effects. All participants read this section before proceeding, given esketamine’s limited use and recognition in Saudi Arabia. Moreover, the research team developed these materials based on what is known about esketamine in the literature (37, 38).

The research team developed the questionnaire to capture the participants’ sociodemographic characteristics (e.g., age, gender, comorbid mental illnesses, current psychotropic medications, and any previous experience with esketamine). Additionally, the questionnaire also entailed questions related to esketamine: (1) transportation, (2) administration setting, (3) medication-related concerns, and (4) depression-related concerns. Notably, we developed the questionnaire items based on the medication’s leaflet information, existing literature on esketamine, and relevant Saudi-specific contextual considerations that the research team deemed relevant.

The MARS-10 is a self-administered, 10-item questionnaire to identify patient adherence to current medications (39, 40). The scale ranges from 0 to 10 (41). The MARS-10 reliability is evidenced by a Cronbach’s α coefficient of 0.75 (42). In the current study, the research team used the scale’s Arabic version after obtaining permission from its authors. The scale’s Arabic version was found to be reliable with a Cronbach’s α coefficient of 0.7 in psychiatric research assessing the adherence of schizophrenic patients (43) and 0.89 in a study assessing the adherence of medication among patients with chronic illnesses such as hypertension and diabetes mellitus (44). Consistent with previous studies, patients with a score ≤ 5 were classified as non-compliant, while those with a score > 5 were classified as compliant (45–48).

### Ethical considerations

The Institutional Review Board of the College of Medicine at King Saud University (research project no. E-24-9385) approved the study. Participants reviewed an electronic informed-consent statement and selected “Next” to access the survey. The survey explained confidentiality, data anonymity, and information regarding the study’s scope and the principal investigator’s contact information. Consent to participate was indicated by clicking on the survey link.

### Statistical data analysis

Data were analyzed using the Statistical Package for the Social Sciences (SPSS, v.26; IBM Corp., Chicago, IL, USA). Descriptive statistics summarized sociodemographic and clinical characteristics, as well as responses to the questionnaire and MARS-10 scale. To explore differences in medication adherence scores, independent samples t-tests compared means between two groups (e.g., presence vs. absence of comorbid mental illness, transportation availability vs. non-availability). One-way analysis of variance (ANOVA) compared adherence scores across groups (e.g., age categories, number of psychiatric medications, and types of comorbid psychiatric conditions). Associations between categorical variables (e.g., adherence status [compliant vs. non-compliant] and sociodemographic/clinical characteristics) were assessed using chi-square tests. Finally, a multiple linear regression analysis identified predictors of medication adherence scores. Independent variables included age, gender, presence of other mental illness, number of psychiatric medications, previous esketamine use, transportation availability, social support (i.e., the availability of someone to take the patient to the hospital, stay with them during/after therapy, or drive them home), cost concerns, and health insurance status. These variables were selected based on prior literature linking them to medication adherence among psychiatric patients. A statistical significance threshold was set at *p* ≤.05.

## Results

Baseline sociodemographic and clinical characteristics of the patients appear in **Table 1**. The sample included 283 patients with depression: 78% female (*n* = 221) and 21.9% male (*n* = 62). Age distribution was 18–25 years, 17.3% (*n* = 49); 26–35, 15.9% (*n* = 45); 36–45, 23.7% (*n* = 67); 46–55, 23.0% (*n* = 65); and 56–65, 20.1% (*n* = 57).

**Table 1:** Baseline sociodemographic and clinical characteristics of the patients (*N* = 283).

Variable	Number (*n*)	Percentage (%)
Gender		
Male	62	21.9
Female	221	78.1
Age group		
18–25 years	49	17.3
26–35 years	45	15.9
36–45 years	67	23.7
46–55 years	65	23.0
56–65 years	57	20.1
Have you ever been diagnosed with any mental illness other than depression?		
No	188	66.4
Yes	95	33.6
Type of other mental illness (*n* = 95)*		
Anxiety disorder	87	91.58
Personality disorders	6	6.32
PTSD	2	2.11
OCD	5	5.26
ADHD	3	3.16
Eating disorders	1	1.05
Others, not specified by the participants	3	3.16
How many psychiatric medications do you currently take regularly (not as needed)?		
None	49	17.3
1 medication	136	48.1
2–3 medications	87	30.7
More than 3	11	3.9
Have you ever taken esketamine before?		
No	252	89.0
Yes	31	11.0
Do you have transportation to get to the hospital?		
No	100	35.3
Yes	183	64.7
Do you have someone (e.g., a relative or friend) who can take you to the hospital?		
No	77	27.2
Yes	206	72.8
Do you have someone (e.g., a relative or friend) who can stay with you during and after the therapy session?		
No	109	38.5
Yes	174	61.5
Do you have someone (e.g., a relative or friend) who can drive you home from the hospital after you get your medication?		
No	132	46.6
Yes	151	53.4
Would you feel stigmatized for using esketamine?		
No	214	75.6
Yes	69	24.4
Will the cost of esketamine (if it is not available at the hospital) be an obstacle to your agreement to take it?		
No	58	20.5
Yes	225	79.5
Do you have health insurance?		
No	223	78.8
Yes	60	21.2

* Patients could choose more than one option.

A third (33.6%, *n* = 95) of participants reported a diagnosis of another mental illness in addition to depression; the remaining two-thirds (66.4%, *n* = 188) did not. Among those with comorbid mental illnesses (*n* = 95, multiple responses allowed), anxiety disorder was most frequent (91.58%, *n* = 87), followed by personality disorders (6.32%, *n* = 6), obsessive-compulsive disorder (5.26%, *n* = 5), attention-deficit/hyperactivity disorder (3.16%, *n* = 3), unspecified other conditions (3.16%, *n* = 3), post-traumatic stress disorder (2.11%, *n* = 2), and eating disorders (1.05%, *n* = 1). Current psychiatric medication use was as follows: one medication, 48.1% (*n* = 136); two to three medications, 30.7% (*n* = 87); more than three medications, 3.9% (*n* = 11); none, 17.3% (*n* = 49).

Only 11.0% (*n* = 31) of the participants had ever used esketamine, whereas 89.0% (*n* = 252) had not. Regarding transportation access (noting that the research team asked about transportation in general without specifying the means of transportation, such as public, private, or both), 64.7% (*n* = 183) reported having access, while 35.3% (*n* = 100) did not.

Regarding social support, 72.8% (*n* = 206) had someone (e.g., a relative or friend) who could take them to the hospital; 61.5% (*n* = 174) had someone available to stay with them during and after therapy. Additionally, 53.4% (*n* = 151) had someone to drive them home after receiving the medication, while 46.6% (*n* = 132) did not.

Regarding perceived stigma, 24.4% (*n* = 69) of participants reported perceived stigma for using esketamine, while 75.6% (*n* = 214) reported no concern about stigma.

Financial considerations were also explored. A large proportion (79.5%, *n* = 225) stated that the cost of esketamine, if unavailable at the hospital, would be an obstacle to their agreement to use it. Finally, 78.8% (*n* = 223) of participants did not have health insurance, while 21.2% (*n* = 60) did.

### Concerns preventing esketamine use

Participants identified several potential barriers to their acceptance or use of esketamine treatment (**Table 2**). Place-related concerns included the need to come to the hospital for each therapeutic dose (45.9%; *n* = 130). Similarly, 45.9% (*n* = 130) were concerned about the requirement for a two-hour post-dose observation. Additionally, 25.4% (*n* = 72) noted a need for transportation to facilitate hospital visits, while 26.9% (*n* = 76) reported needing someone to accompany them to the hospital. Approximately one-third (32.5%, *n* = 92) stated the need for someone to stay with them during and after the therapy session, and 26.1% (*n* = 74) mentioned the need for someone to take them home after receiving the medication. Despite these concerns, 33.2% (*n* = 94) reported no place-related concerns (**Table 2**).

**Table 2:** Concerns preventing esketamine use as perceived by the patients (*N* = 283).

Concern	Number (*n*)	Percentage (%)
Place-related concerns		
It is necessary to come to the hospital to take the therapeutic dose	130	45.9
Provides transportation to facilitate your arrival to the hospital	72	25.4
Have someone (e.g., a relative or friend) available to take you to the hospital	76	26.9
Have someone (e.g., a relative or friend) available who can stay with you during and after the therapy session	92	32.5
Have someone available to take you home from the hospital after you receive your medication	74	26.1
The need to remain under medical observation for two hours after receiving the dose of the medication	130	45.9
No concerns about where to take the medication	94	33.2
Medication-related concerns		
Feeling stigmatized for using such a medication	63	22.3
Fears of unavailability or discontinuity of medication	169	59.7
Cost of medication (if applicable, if not available at the hospital)	184	65.0
How to take the medicine (the medicine is taken as a nasal spray)	55	19.4
The need to take the medication twice a week in the first month of treatment	60	21.2
Not knowing enough about the medicine	129	45.6
Side effects of the medicine	163	57.6
Fears of drug addiction	143	50.5
No concerns about the medication	35	12.4
Depression-related concerns		
The number of psychiatric medications you have tried without benefit	100	35.3
The number of psychiatric medications you currently take regularly	81	28.6
Severity of depression	76	26.9
Years being diagnosed with depression	110	38.9
No concerns related to depression	97	34.3

Regarding medication-related concerns, 65.0% (*n* = 184) cited medication cost (if unavailable at the hospital) as a barrier, and 59.7% (*n* = 169) expressed fear about the unavailability or discontinuity of the medication. A majority of participants (57.6%; *n* = 163) reported concerns about potential side effects, while 50.5% (*n* = 143) feared the possibility addiction. A notable minority (45.6%; *n* = 129) cited a lack of sufficient knowledge about the medication. Other concerns included feeling stigmatized for using esketamine (22.3%, *n* = 63), concern about how to take the medication (nasal spray format; 19.4%, *n* = 55), and the need to take it twice per week during the first month of treatment (21.2%, *n* = 60). Only 12.4% (*n* = 35) reported no medication-related concerns (**Table 2**).

Regarding depression-related concerns, 35.3% (*n* = 100) reported that the number of previously tried psychiatric medications without benefit was a concern. Additionally, 28.6% (*n* = 81) expressed concern about the number of psychiatric medications they were currently taking. Depression severity was a concern for 26.9% (*n* = 76), while 38.9% (*n* = 110) indicated that the number of years since being diagnosed with depression contributed to their hesitation. Nonetheless, 34.3% (*n* = 97) reported having no depression-related concerns when considering esketamine use (**Table 2**).

### Acceptance and preferences regarding esketamine

When asked whether the dosing schedule of esketamine—once or twice a week—would make them prefer it over other daily antidepressants, 51.2% (*n* = 145) of participants responded affirmatively, while 48.8% (*n* = 138) did not express a preference based on this characteristic (**Table 3**).

**Table 3:** Depression patients’ acceptance and preferences regarding taking esketamine (*N* = 283).

Question	Number (*n*)	Percentage (%)
Considering that esketamine is taken once or twice a week, would this characteristic make you prefer it over other daily antidepressants?		
No	138	48.8
Yes	145	51.2
Taking into account all the educational information about esketamine provided at the beginning of the questionnaire, would you agree to take esketamine if your doctor advised you to start it to treat your condition?		
No	135	47.7
Yes	148	52.3

After reviewing the educational information about esketamine provided at the beginning of the survey, 52.3% (*n* = 148) of participants would agree to take esketamine if their doctor advised them to do so to treat their condition. In contrast, 47.7% (*n* = 135) indicated that they would not agree to take it even with medical advice (**Table 3**).

### Responses to MARS-10 items

Among the 234 participants taking one or more psychiatric medications, MARS-10 responses reflected varied adherence behaviors (**Table 4**). Most participants (71.4%, *n* = 167) sometimes forgot to take their medication, while 28.6% (*n* = 67) did not forget. About one-third (35.5%, *n* = 83) admitted to being occasionally careless about taking their medication, while 64.5% (*n* = 151) disagreed (**Table 4**).

**Table 4:** Depression patients’ responses to MARS-10 scale items (*n* = 234).

Item	Yes *n* (%)	No *n* (%)
1. Do you ever forget to take your medication?	167 (71.4%)	67 (28.6%)
2. Are you careless at times about taking your medicine?	83 (35.5%)	151 (64.5%)
3. When you feel better, do you sometimes stop taking your medicine?	42 (17.9%)	192 (82.1%)
4. Sometimes if you feel worse when you take the medicine, do you stop taking it?	44 (18.8%)	190 (81.2%)
5. I take my medication only when I am sick.	51 (21.8%)	183 (78.2%)
6. It is unnatural for my mind and body to be controlled by medication.	92 (39.3%)	142 (60.7%)
7. My thoughts are clearer on medication.	162 (69.2%)	72 (30.8%)
8. By staying on medication, I can prevent getting sick.	174 (74.4%)	60 (25.6%)
9. I feel weird, like a “zombie,” on medication.	34 (14.5%)	200 (85.5%)
10. Medication makes me feel tired and sluggish.	108 (46.2%)	126 (53.8%)

When asked about ceasing medication when feeling better, only 17.9% (*n* = 42) answered “Yes,” while most (82.1%, *n* = 192) continued their medication. Similarly, 18.8% (*n* = 44) reported stopping medication when they feel worse, and 21.8% (*n* = 51) stated that they take their medication only when sick (**Table 4**).

Regarding beliefs about medication, 39.3% (*n* = 92) felt that it was unnatural for medication to control their mind and body. However, a strong majority (69.2%, *n* = 162) believed their thoughts were clearer while on medication, and 74.4% (*n* = 174) agreed that staying on medication helps prevent illness (**Table 4**).

Few patients (14.5%, *n* = 34) reported feeling like a “zombie” on medication, with most (85.5%, *n* = 200) denying this experience. Additionally, 46.2% (*n* = 108) felt tired or sluggish from their medication, while 53.8% (*n* = 126) did not share this sentiment (**Table 4**).

### Medication adherence levels

Based on the responses to the MARS-10 scale, 77.4% (*n* = 181) were non-compliant, and 22.6% (*n* = 53) were compliant with their current medications (**Table 5**).

**Table 5:** Levels of medication adherence among depression patients based on MARS-10 (*n* = 234).

Level of medication adherence	Number (*n*)*	Percentage (%)
Non-compliant (total score ≤5)	181	77.4%
Compliant (total score >5)	53	22.6%
Total	234	100.0%

* Based on a sample size of 234 (participants taking one medication or more).

### Differences in adherence scores by patient characteristics

An independent samples *t*-test examined whether medication adherence scores (as measured by the MARS-10) differed significantly across various patient characteristics (**Table 6**). There were no significant differences in adherence scores based on gender, *t*(281) = −0.47, *p* = .636, or transportation availability to the hospital, *t*(281) = 0.15, *p* = .878. Additionally, medication adherence did not significantly differ between participants with health insurance and those without, *t*(281) = −0.31, *p* = .759, nor between those who reported stigma and those who did not, *t*(281) = −0.61, *p* = .544 (**Table 6**).

**Table 6:** Independent samples *t*-test for differences in medication adherence based on the depression patients’ characteristics (*n* = 234).

Variable	Mean ± *SD*	*t*	*P*-value
Gender			
Male	3.96 ± 2.24		
Female	4.12 ± 2.13	−0.473	.636
Have you ever been diagnosed with any mental illness other than depression?			
No	3.91 ± 2.16		
Yes	4.41 ± 2.12	−1.691	.092
Have you ever taken esketamine before?			
No	3.87 ± 2.03		
Yes	5.78 ± 2.34	−4.505	.000*
Do you have transportation to get to the hospital?			
No	4.13 ± 2.45		
Yes	4.08 ± 2.04	0.153	.878
Do you have someone (e.g., a relative or friend) who can take you to the hospital?			
No	3.79 ± 2.07		
Yes	4.28 ± 2.19	−1.700	.090
Do you have someone (e.g., a relative or friend) who can stay with you during and after the therapy session?			
No	3.78 ± 1.90		
Yes	4.34 ± 2.31	−1.995	.047*
Do you have someone (e.g., a relative or friend) who can drive you home from the hospital after you get your medication?			
No	3.86 ± 2.01		
Yes	4.22 ± 2.22	−1.235	.218
Would you feel stigmatized for using such a drug?			
No	4.04 ± 2.12		
Yes	4.24 ± 2.27	−0.608	.544
Will the cost of the medication (if it is not available at the hospital) be an obstacle to your agreement to take it?			
No	3.46 ± 2.46		
Yes	4.24 ± 2.05	−2.244	.026*
Do you have health insurance?			
No	4.07 ± 2.06		
Yes	4.17 ± 2.48	−0.307	.759

* Significant at significance level *p* ≤ 0.05.

However, three variables revealed statistically significant differences. Prior esketamine use was linked to significantly higher adherence scores (*M* = 5.78, *SD* = 2.34) versus no prior use (*M* = 3.87, *SD* = 2.03), *t*(281) = −4.51, *p* <.001. Having someone to stay during and after therapy was associated with higher adherence (*M* = 4.34, *SD* = 2.31) than not having such support (*M* = 3.78, *SD* = 1.90), *t*(281) = −2.00, *p* = .047. Furthermore, individuals who reported cost as a barrier had significantly higher adherence scores (*M* = 4.24, *SD* = 2.05) than those who did not report cost concerns (*M* = 3.46, *SD* = 2.46), *t*(281) = −2.24, *p* = .026 (**Table 6**).

Other comparisons, including having a mental illness other than depression (*p* = .092), having someone to take the patient to the hospital (*p* = .090), and having someone to drive the patient home after medication (*p* = .218), did not exhibit statistically significant differences (**Table 6**).

A one-way ANOVA assessed adherence based on age group, type of psychiatric diagnosis (in patients with comorbid conditions), and number of psychiatric medications currently taken (**Table 7**). The results indicated no significant difference in adherence scores across age groups, *F*(4, 278) = 0.19, *p* = .943. Participants aged 18–25 years reported the highest mean adherence score (*M* = 4.31, *SD* = 2.02), while those aged 36–45 years reported the lowest (*M* = 3.98, *SD* = 2.02); however, these differences were not statistically significant (**Table 7**).

**Table 7:** One-way analysis of variance (ANOVA) test for differences in medication adherence based on the depression patients’ characteristics (*n* = 234).

Variable	Mean ± *SD*	*F*-value	*p*-value
Age group			
18–25 years	4.31 ± 2.02		
26–35 years	4.25 ± 2.45		
36–45 years	3.98 ± 2.02		
46–55 years	4.02 ± 2.40		
56–65 years	4.04 ± 1.90	0.191	.943
Psychiatric diagnoses			
Anxiety	4.07 ± 2.17		
Personality disorders	5.00 ± 2.10		
PTSD	6.00 ± 0.00		
Obsessive-compulsive disorder	3.40 ± 2.80		
Attention-deficit/hyperactivity disorder	4.00 ± 1.41		
Eating disorders	3.00 ± 0.00		
Others, not specified by the participants	5.33 ± 1.53	0.733	.612
Number of psychiatric medications			
1 medication	3.95 ± 2.14		
2–3 medications	4.25 ± 2.22		
More than 3 medications	4.55 ± 1.86	0.786	.457

Similarly, adherence did not significantly differ based on comorbid psychiatric diagnosis, *F*(6, 100) = 0.73, *p* = .612. Patients with PTSD (*M* = 6.00, *SD* = 0.00) and those with unspecified conditions (*M* = 5.33, *SD* = 1.53) had numerically higher adherence scores than other subgroups; however, these differences did not reach statistical significance (**Table 7**).

Adherence also did not significantly differ in terms of the number of psychiatric medications currently taken, *F*(2, 280) = 0.79, *p* = .457. Patients taking more than three medications had the highest mean adherence (*M* = 4.55, *SD* = 1.86), while those on one medication had the lowest (*M* = 3.95, *SD* = 2.14), though these differences were not statistically meaningful (**Table 7**).

### Associations between adherence and patient characteristics

Chi-square tests examined associations between medication adherence status (compliant vs. non-compliant) and various sociodemographic and clinical characteristics among patients with depression (**Table 8**). No statistically significant associations between medication adherence and gender emerged, χ²(1, *N* = 234) = 0.11, *p* = .451; age group, χ²(4, *N* = 234) = 3.23, *p* = .520; presence of a mental illness other than depression, χ²(1, *N* = 234) = 1.09, *p* = .296; number of psychiatric medications taken regularly, χ²(2, *N* = 234) = 1.45, *p* = .483; having someone to transport the patient to the hospital, χ²(1, *N* = 234) = 2.39, *p* = .122; having someone to drive them home, χ²(1, *N* = 234) = 1.54, *p* = .215; stigma perception, χ²(1, *N* = 234) = 0.35, *p* = .556; cost concerns, χ²(1, *N* = 234) = 0.31, *p* = .577; or health insurance status, χ²(1, *N* = 234) = 0.56, *p* = .456 (**Table 8**).

**Table 8:** Differences in the levels of medication adherence based on the depression patients’ characteristics (*n* = 234).

Variable	Non-compliant (*n* = 181)	Compliant (*n* = 53)	χ^2^	*p*-value
Gender				
Male	38 (21%)	10 (18.9%)	0.114	.451
Female	143 (79%)	43 (81.1%)		
Age				
18–25 years	24 (13.3%)	8 (15.1%)	3.232	.520
26–35 years	25 (13.8%)	11 (20.8%)		
36–45 years	46 (25.4%)	9 (17%)		
46–55 years	44 (24.3%)	15 (28.3%)		
56–65 years	42 (23.2%)	10 (18.9%)		
Have you ever been diagnosed with any mental illness other than depression?				
No	120 (66.3%)	31 (58.5%)	1.092	.296
Yes	61 (33.7%)	22 (41.5%)		
How many psychiatric medications do you currently take regularly (not as needed)?				
1 medication	109 (60.2%)	27 (50.9%)	1.454	.483
2–3 medications	64 (35.4%)	23 (43.4%)		
More than 3 medications	8 (4.4%)	3 (5.7%)		
Have you ever taken esketamine before?				
No	167 (92.3%)	40 (75.5%)	11.327	.001*
Yes	14 (7.7%)	13 (24.5%)		
Do you have transportation to get to the hospital?				
No	44 (24.3%)	20 (37.7%)	3.719	.054*
Yes	137 (75.7%)	33 (62.3%)		
Do you have someone (e.g., a relative or friend) who can take you to the hospital?				
No	76 (42%)	16 (30.2%)	2.393	.122
Yes	105 (58%)	37 (69.8%)		
Do you have someone (e.g., a relative or friend) who can stay with you during and after the therapy session?				
No	89 (49.2%)	17 (32.1%)	4.835	.028*
Yes	92 (50.8%)	36 (67.9%)		
Do you have someone (e.g., a relative or friend) who can drive you home from the hospital after you get your medication?				
No	68 (37.6%)	15 (28.3%)	1.538	.215
Yes	113 (62.4%)	38 (71.7%)		
Would you feel stigmatized for using such a drug?				
No	137 (75.7%)	38 (71.7%)	0.347	.556
Yes	44 (24.3%)	15 (28.3%)		
Will the cost of the medication (if it is not available at the hospital) be an obstacle to your agreement to take it?				
No	37 (20.4%)	9 (17%)	0.311	.577
Yes	144 (79.6%)	44 (83%)		
Do you have health insurance?				
No	142 (78.5%)	39 (73.6%)	0.555	.456
Yes	39 (21.5%)	14 (26.4%)		

* Significant at significance level *p* ≤.05.

However, three variables were significant or near-significant. Patients who had previously taken esketamine were significantly more likely to be classified as adherent (24.5%) than non-adherent patients (7.7%), χ²(1, *N* = 234) = 11.33, *p* = .001 (**Table 8**). Similarly, having someone available to stay with the patient during and after therapy was significantly associated with adherence status. Among compliant patients, 67.9% had such support compared to 50.8% of non-compliant patients, χ²(1, *N* = 234) = 4.84, *p* = .028 (**Table 8**). Additionally, transportation availability approached statistical significance: a higher proportion of compliant patients lacked transportation (37.7%) compared to non-compliant patients (24.3%), χ²(1, *N* = 234) = 3.72, *p* = .054 (**Table 8**).

### Predictors of medication adherence

A multiple linear regression analysis examined whether various demographic and clinical factors significantly predicted medication adherence scores among depression patients (**Table 9**). The model was statistically significant, *F*(10, 223) = 2.27, *p* <.05, and explained a substantial portion of the variance in medication adherence, *R* = .979, *R*² = .958, adjusted *R*² = .535. The standard error of the estimate was 1.43.

**Table 9:** Predictors of medication adherence among depression patients (*n* = 234).

Model	Unstandardized coefficients		Standardized coefficients	*t*	Sig.	
	B	Std. error	Beta			
(Constant)	2.171	6.876		.316	.805	
Gender	4.714	3.677	.872	1.282	.422	
Age	−1.571	1.271	−.962	−1.236	.433	
Other mental illnesses	−.457	.840	−.397	−.544	.683	
How many psychiatric medications do you currently take regularly (not as needed)?	1.657	4.957	.406	.334	.795	
Have you ever taken esketamine before?	−1.343	3.641	−.184	−.369	.775	
Do you have transportation to get to the hospital?	−4.000	5.367	−.549	−.745	.592	
Do you have someone (e.g., a relative or friend) who can take you to the hospital?	−1.086	4.506	−.149	−.241	.849	
Do you have someone (e.g., a relative or friend) who can stay with you during and after the therapy session?	1.257	3.752	.233	.335	.794	
Will the cost of the medication (if it is not available at the hospital) be an obstacle to your agreement to take it?	−.057	2.242	−.008	−.025	.984	
Do you have health insurance?	1.200	3.740	.258	.321	.802	
a. Dependent variable: medication adherence					

The following variables were excluded from the model due to multicollinearity or non-significance: “Do you have someone (e.g., a relative or friend) who can drive you home from the hospital after you get your medication?” and “Would you feel stigmatized for using such a drug?”.

Despite the overall model reaching statistical significance, none of the individual predictors were statistically significant. Gender (β = .872, *p* = .422), age (β = –.962, *p* = .433), and having other mental illnesses (β = −.397, *p* = .683) did not significantly predict adherence. Similarly, the number of psychiatric medications taken (β = .406, *p* = .795), prior esketamine use (β = −.184, *p* = .775), and transportation availability (β = −.549, *p* = .592) were not significant (**Table 9**).

Additional factors, including having someone to take the patient to the hospital (β = −.149, *p* = .849), someone to stay with patients during and after therapy (β = .233, *p* = .794), cost as a barrier (β = −.008, *p* = .984), and health insurance (β = .258, *p* = .802), were also not significantly associated with adherence levels (**Table 9**).

## Discussion

Approximately one-third of participants reported a comorbid mental illness, while two-thirds did not. Among those with comorbidity, anxiety disorder was the most common. This result aligns with other studies, such as a study from the Netherlands (49) and another from Australia (50), both indicating that anxiety is the most common comorbidity among patients with depression. Further, this high depression-anxiety comorbidity has been linked to more severe depressive symptoms (49), poorer treatment response (49), and increased risk of relapse (49). Therefore, recognizing this depression-anxiety comorbidity is critical, as early detection and simultaneous management of both conditions could significantly improve clinical outcomes in this population. Such a finding reflects the importance of routine screening for other mental illnesses, especially anxiety disorders, in this particular population.

In this study, financial considerations emerged as a significant barrier when considering esketamine treatment, with over three-quarters of the participants (79.5%) reporting esketamine’s cost as a potential barrier to use. This finding aligns with a U.S study (51), which reported that esketamine’s cost is a significant barrier to its use, finding that esketamine would only be cost-effective if the per-dose price were reduced by more than 40%. In another U.S. study (52), cost and insurance emerged as potential barriers to esketamine use. In Saudi Arabia, the single nasal spray bottle of esketamine costs around 952.90 SAR (53), which is considered high, suggesting that financial concerns may discourage treatment initiation (54) and limit accessibility and equitable use. Additionally, limited insurance coverage may further exacerbate these challenges in Saudi Arabia. The finding regarding patient perceptions of cost as a major barrier highlights the importance of establishing transparent pricing structures, expanding insurance coverage, and implementing sustainable reimbursement strategies to facilitate wider access to esketamine for Saudi patients with depression. Future research should evaluate financial models and policy interventions that reduce cost-related barriers, ensuring equitable access and improving adherence to esketamine treatment.

Nearly half of this study’s participants (45.9%) cited the need for hospital visits and a two-hour observation after the esketamine dose as deterrents. This result is consistent with other studies, such as one from the United Kingdom (31) and another from the U.S (55). These studies (31, 55) collectively affirm that mandatory therapy attendance and extended post-dose monitoring present significant barriers to esketamine treatment. Accordingly, to help patients overcome such a barrier, practical supportive measures could be considered (31, 55). Such measures could include providing transportation assistance, scheduling flexible therapy session times, and involving family members or caregivers to accompany patients, all of which may make attending more feasible (31, 55). Future research should also investigate the effect of such measures in reducing the logistical burden of esketamine treatment.

In this study, more than half (57.6%) of the patients reported concerns about potential side effects of esketamine treatment. These results align with studies conducted in China (23, 56), which found that concerns about side effects can directly affect treatment adherence, often leading to hesitation or discontinuation of esketamine therapy. Further, these studies (23, 56) found that commonly reported adverse effects among participants include dissociation, dizziness, nausea, and transient increases in blood pressure. Future research should explore ways to improve pre-treatment psychoeducation to help improve patients’ confidence and willingness to continue therapy, as well as practical strategies to manage specific side effects and make treatment easier to tolerate, ultimately supporting better adherence.

Moreover, about half of this study’s patients prefer to take esketamine once or twice a week over other daily antidepressants. This finding aligns with a U.S. study (57) that indicated a preference for esketamine treatment compared to the slower daily oral antidepressants. The U.S. study (57) further indicated that treatments with less-frequent dosing and faster onsets, such as esketamine, are generally better tolerated and more acceptable to patients compared to daily oral antidepressants. Future studies should examine approaches that prioritize patient convenience by optimizing dosing schedules and supporting adherence, particularly for therapies with less-frequent administration and rapid onset.

The present study found that about half (52.3%) of the patients would agree to take esketamine under a doctor’s advisement to treat their condition, while the remaining indicated they would not agree to take it even with medical advice. This result aligns with another U.S. study (58) that found that over half of the participants were likely to follow a physician’s advice to take esketamine, with the other half refusing to initiate treatment even with a medical recommendation. Future studies should investigate the effectiveness of structured psychoeducation programs in addressing patient hesitancy toward esketamine, particularly for those who may refuse treatment even when recommended by a physician (59). Such programs could include counseling on treatment benefits, side-effect management, and the expected course of therapy.

Moreover, in this study, the majority of participants (71.4%) reported that they sometimes forget to take medication. This finding corresponds to a U.S. study (60), which found that simply forgetting was the main reason for patient non-adherence. Additionally, another Canadian study (61) reported that forgetfulness was the most common reason for noncompliance. Given that forgetfulness is a frequently reported issue in patients’ adherence, we recommend the routine use of reminders, such as phone alarms and medication apps, as well as family involvement, to support adherence to antidepressant treatment.

In this sample, over three-quarters of patients with depression (77.4%) were non-adherent to their current psychiatric medications, exceeding the more than half (~50%) non-adherence rates commonly reported among international psychiatric populations (31, 34). Several factors may underlie this study’s elevated non-adherence rate, including forgetfulness and lack of illness insight, which are frequently cited in the literature as barriers to regular medication intake (60, 61). In addition, stigma associated with psychiatric disorders, limited health literacy, and negative beliefs about psychotropic medications can all contribute to non-adherence (35). In the Saudi context, cultural dynamics may also play a role, such as patients sometimes relying more on family decision-making or traditional beliefs, which may interfere with long-term adherence (35). Together, these elements highlight the multifaceted nature of poor adherence in patients with depression, highlighting the imperative to employ comprehensive interventions. Structured education programs, consistent follow-up visits, and motivational interviewing approaches can help overcome these barriers, while digital reminders and family-based support strategies may address practical issues such as forgetfulness.

The findings revealed higher medication adherence among patients with prior esketamine exposure and among patients with someone to accompany them to and from therapy sessions. These findings align with past research that has demonstrated the positive impact of treatment experience and support systems on adherence to psychiatric medication (31, 34). Importantly, we hypothesize that this difference is unlikely to be explained by a lingering pharmacological effect of esketamine. Rather, we hypothesize that it reflects the influence of treatment experience and perceived benefit—factors that prior studies have emphasized as key adherence drivers (31, 34). Another plausible explanation is that these patients were more likely to have TRD, had experienced genuine symptom improvement with esketamine, and were, therefore, more motivated to maintain care. Experiencing benefit from an otherwise treatment-resistant illness may have strengthened patients’ belief in the value of psychiatric care, which in turn reinforced adherence. Esketamine trial data support this explanation. For instance, Pepe et al. (34) found that patients who responded to intranasal esketamine were more likely to demonstrate long-term adherence and emphasized the predictive value of treatment familiarity and the perceived benefit of adherence behavior. Similarly, qualitative research on ketamine has established a link between perceived treatment benefits and a motivation to continue (31). At the same time, another study cautions that treatment adherence in TRD can be low despite exposure to new treatments, given the chronic and relapsing nature of the illness (62). Accordingly, future research should use longitudinal designs and large, diverse samples to determine whether the higher adherence observed in this study’s patients regarding esketamine exposure can be sustained over the long term.

Additionally, social and logistical support were critical factors in medication adherence in this study. Having a companion during treatment sessions or for the trip home improved adherence, and transportation availability also approached statistical significance as an adherence predictor. These findings support previous research demonstrating that family support and logistical facilitation are key determinants of adherence in psychiatric care (31, 34). The logistical aspect is also noteworthy: transportation difficulties may limit patients’ ability to attend regular treatment sessions, especially in cases where treatment requires frequent hospital visits, such as with esketamine. In Saudi Arabia, where travel distances can be considerable and public transportation is limited, transportation barriers are particularly relevant. Therefore, addressing transportation barriers—through healthcare-provided transport services, community initiatives, or insurance coverage—may significantly improve adherence. In addition, we recommend that future care plans proactively involve family members in ongoing support, as well as to overcome logistical barriers as part of a comprehensive adherence-enhancing strategy.

Regarding demographic and clinical factors using ANOVA, this study found no significant differences in medication adherence based on age, type of psychiatric comorbidity, or the number of medications taken. This finding aligns with earlier studies, which reported that younger age and fewer medications are associated with better adherence (29, 30) and that certain psychiatric comorbidities, such as anxiety and obsessive-compulsive disorder, are often linked to lower adherence (62). One possible explanation is that strong family involvement in the Saudi population may buffer the effects of age or comorbidity, leading to a more uniform adherence pattern across demographic subgroups. Future qualitative Saudi studies are needed to gain better insight into these probable underlying factors.

Finally, although the regression model was statistically significant overall, no individual predictor—such as gender, age, social support, insurance status, or history of esketamine use—was significant. This finding diverges from previous reports where demographic and psychosocial variables were noted to predict adherence (29, 30). Therefore, further Saudi research should be conducted to compare results in the Saudi context.

### Strengths and limitations

This study has several strengths. It is among the first in Saudi Arabia to investigate esketamine and patient perceptions, addressing a critical gap in the regional literature. This study also examined barriers and obstacles that patients could face during esketamine treatment, providing a foundation for future research and interventions and potentially leading to the successful adoption of esketamine treatment in psychiatric care. In addition, the sample size was sufficient for statistically meaningful conclusions.

However, this study also has limitations. First, the cross-sectional design precludes a direct causal inference. Hence, future Saudi studies using a longitudinal design to better explore causal relationships over time are warranted. Second, self-reported data are susceptible to recall issues or participants giving socially desirable answers, requiring the consideration of objective measures or combining self-reports with clinician assessments to improve accuracy. For instance, comorbid psychiatric conditions were self-reported, which may have led to the underestimation or overestimation of the actual figures; future studies should use more rigorous methods to assess such comorbidities, such as corroborating with medical records or clinical assessments. Third, although the research team phrased the study tool questions as clearly as possible, some participant responses should be interpreted with caution, as participants may vary in their understanding of questions, potentially leading to misleading answers and, hence, results. For example, regarding the question asking the participants whether the severity of depression was a concern, some participants might have understood that their depression was too severe to respond to treatment, while others might have understood that their depression was mild; therefore, they do not need a newer treatment such as esketamine. Fourth, specific questions in the study merit greater specificity and analytic depth, which future studies could explore to inform the development of targeted interventions. For instance, in this study, questions related to transportation were phrased without specifying the availability of public, private, or both public and private means of transportation. Exploring these various modes of transportation in more depth and developing interventions to facilitate transportation to receive the esketamine could help overcome the transportation-related concern. Additionally, regarding the study tools, another limitation is that the study did not employ a standardized scale to assess depression diagnosis and its severity, as the study’s aim was to evaluate the perception and attitude toward esketamine among those already known to suffer from depression, regardless of the severity of the illness, noting that the participants were being followed in psychiatric clinics and were already diagnosed with depression. However, future Saudi research should consider using structured or semi-structured interviews or a standardized scale to further confirm the accuracy and severity of the illness, as this may lead to more accurate and reflective results. Participant-related constraints further limited generalizability. The sample included participants diagnosed with depression irrespective of TRD status, which is the main indication for using esketamine per international guidelines (63). Thus, this study’s participants may not fully reflect those who benefit from esketamine, namely those with TRD. Therefore, future studies in Saudi Arabia should specifically target populations relevant to esketamine usage. Another participant-related limitation is that the sample may not fully reflect the broader population of patients with depression in Saudi Arabia, thereby limiting the generalizability of the findings. Including participants from different regions and healthcare settings would help improve representativeness.

## Conclusion

This study aimed to assess the awareness and acceptance of esketamine as a treatment option and explored factors potentially influencing acceptance among patients with depression. Key barriers regarding the acceptance of esketamine included the need to come to the hospital and undergo observation after treatment. To overcome these concerns, we suggest providing social support to the patients to facilitate their acceptance of the medication. Another concern was the cost associated with esketamine. To reduce the cost-related concern, we recommend that insurance cover esketamine and that government hospitals make it readily available or perhaps support its local production, if feasible. Furthermore, this study found that patients’ adherence to their current medication regimen was significantly associated with having previous esketamine experience. Therefore, we recommend providing the patients with related educational material to improve their awareness and, hence, facilitate their acceptance of esketamine as a therapeutic option when clinically indicated. Furthermore, social support was associated with adherence to the current medication regimen. This reflects the importance of multidisciplinary team involvement, including social workers, to provide the needed social support to patients and their families. Collectively, these strategies may enhance equitable access, acceptance, and adherence within the Saudi context.

## Data Availability

The raw data supporting the conclusions of this article will be made available by the authors, without undue reservation.
